# Scanning Electron Microscopic Findings on Respiratory Organs of Some Naturally Infected Dromedary Camels with the Lineage-B of the Middle East Respiratory Syndrome Coronavirus (MERS-CoV) in Saudi Arabia—2018

**DOI:** 10.3390/pathogens10040420

**Published:** 2021-04-01

**Authors:** Abdelmohsen Alnaeem, Samy Kasem, Ibrahim Qasim, Mohamed Refaat, Ali Nasser Alhufufi, Ali Al-Doweriej, Abdulkareem Al-Shabebi, Abd-El Rahman Taha Hereba, Maged Gomaa Hemida

**Affiliations:** 1 Department of Clinical Studies, College of Veterinary Medicine, King Faisal University, Al-Haa 400, Saudi Arabia; aaalnaeem@kfu.edu.sa; 2 Veterinary Health and Monitoring, Ministry of Environment, Water and Agriculture, Riyadh 11195, Saudi Arabia; samy_kasem1976@yahoo.com (S.K.); Dr.ibrahim@moa.gov.sa (I.Q.); refaatpath@yahoo.com (M.R.); Ali_vet200@hotmail.com (A.N.A.); Dr.alivet@mewa.gov.sa (A.A.-D.); 3 Department of Virology, Faculty of Veterinary Medicine, Kafrelsheikh University, Kafrelsheikh 33516, Egypt; ahereba@kfu.edu.sa; 4 Department of Pathology, Animal Health Research Institute, Dokki, Cairo 12618, Egypt; 5 Department of Anatomy, College of Veterinary Medicine, King Faisal University, Al-Haa 400, Saudi Arabia; Karem_vet@yahoo.com; 6 Department of Microbiology, College of Veterinary Medicine, King Faisal University, Al-Haa 400, Saudi Arabia

**Keywords:** MERS-CoV, dromedary camel, SEM, ciliary loss, lesion scoring

## Abstract

The currently known animal reservoir for MERS-CoV is the dromedary camel. The clinical pattern of the MERS-CoV field infection in dromedary camels is not yet fully studied well. Some pathological changes and the detection of the MERS-CoV antigens by immunohistochemistry have been recently reported. However, the nature of these changes by the scanning electron microscope (SEM) was not revealed. The objective of this study was to document some changes in the respiratory organs induced by the natural MERS-CoV infection using the SEM. We previously identified three positive animals naturally infected with MERS-CoV and two other negative animals. Previous pathological studies on the positive animals showed varying degrees of alterations. MERS-CoV-S and MERS-CoV-Nc proteins were detected in the organs of positive animals. In the current study, we used the same tissues and sections for the SEM examination. We established a histopathology lesion scoring system by the SEM for the nasal turbinate and trachea. Our results showed various degrees of involvement per animal. The main observed characteristic findings are massive ciliary loss, ciliary disorientation, and goblet cell hyperplasia, especially in the respiratory organs, particularly the nasal turbinate and trachea in some animals. The lungs of some affected animals showed signs of marked interstitial pneumonia with damage to the alveolar walls. The partial MERS-CoV-S gene sequencing from the nasal swabs of some dromedary camels admitted to this slaughterhouse confirms the circulating strains belong to clade-B of MERS-CoV. These results confirm the respiratory tropism of the virus and the detection of the virus in the nasal cavity. Further studies are needed to explore the pathological alterations induced by MERS-CoV infection in various body organs of the MERS-CoV naturally infected dromedary camels.

## 1. Introduction

The Middle East respiratory syndrome (MERS-CoV) was identified in 2012 in the Arabian Peninsula [1]. It is well known that the dromedary camels act as one of the major reservoirs of the virus [2]. Previous research studies confirmed the detection and characterization of MERS-CoV mainly from dromedary camels in the Arabian Peninsula [3,4,5,6,7]. Several studies reported the seroconversion of dromedary camels in several African countries [2,3,7,8]. 

Some candidates of the family Camelidae, such as alpaca, and the llama, were found to be susceptible to the experimental as well as the field infection of MERS-CoV [9,10]. One study detected antibodies in sera of some alpaca raised in MERS-CoV endemic areas. Since these animals did not receive any MERS-CoV vaccines, these antibodies most likely resulted from MERS-CoV natural infection [11]. Initially, the MERS-CoV infection in camels was studied under the experimental infection approach [12]. Meanwhile, the details of the virus shedding in dromedary camels under experimental conditions have been reviewed by several research groups [12,13]. In the same manner, the clinical course of MERS-CoV infection in alpaca under an experimental approach has also been reported. These studies suggested both llama and alpaca might act as surrogate animals to study MERS-CoV infection, particularly in countries, which do not have dromedary camels. Another study showed the high prevalence (61.5%) of the MERS-CoV -RNAs in lung tissues of dromedary camels submitted for slaughter in one slaughterhouse in the eastern regions of Saudi Arabia [6]. 

MERS-CoV tropism was studied using both in vivo in cell culture such as Vero cell line and in the ex vivo model using tissues from lung donors [14]. This study confirmed that MERS-CoV can infect the human lung tissues as well as adopt some immune evasion strategies to evade the host immune system halting interferon production [14]. Several studies reported the detection of the MERS-CoV-RNAs in the upper respiratory tract, including the right lobe of the lung [6,12,13]. 

The electron microscope has played essential roles in the identification of some coronaviruses affecting humans and animals [15,16,17]. Both the SEM and the TEM were used to study the tropism and pathological alterations triggered by the severe acute respiratory syndrome coronavirus-2 (SARS-CoV-2) in several organs of infected patients [18]. 

Although there are some studies conducted to show the normal histology of some respiratory organs of dromedary camels [19,20], little is still known about the normal histology of various dromedary camel organs by SEM as well as the pathological impacts of MERS-CoV on dromedary camels under field conditions. Only one study showed a marked ciliary loss of some respiratory organs, especially the nasal turbinates, after being experimentally inoculated with (recombinant MVA expressing MERS-CoV spike protein) [21]. The same study proved the preservation of these ciliary structures of the infected animals previously vaccinated with the Modified-Vaccinia-Virus-Ankara- MERS-CoV-based vaccine (MVA-S) [21]. However, no previous studies showed the alteration in the histology of tissues of some MERS-CoV infected animals under natural field conditions using the SEM approach. This study was conducted to explore the alteration of the respiratory organs in dromedary camels infected with MERS-CoV under field conditions by the SEM. 

## 2. Results

### 2.1. Identification of Some MERS-CoV Field Infected Animals

We screened nasal swabs from 69 dromedary camels with real-time PCR to identify some active MERS-CoV animal shedders. The physical examination of these animals before slaughter showed no obvious clinical respiratory signs [22]. Despite some mild rhinorrhea in a limited number of animals. These animals breathe normally with no apparent signs of tachypnoea. The range of the Ct values of the nasal swabs collected from these animals was from (16–29). Out of these animals, we identified three animals with the lowest Ct values (≤16) to ensure the high viral titers in the respiratory passages of these animals. Meanwhile, we selected two negative MERS-CoV dromedary camels to act as a negative control for the downstream testing of their respiratory organs with the SEM. 

### 2.2. Histopathological and Immunohistochemical Changes in Some Respiratory Organs Obtained from MERS-CoV Field Infected Animals Using the Light Microscope

**Figure 1:**
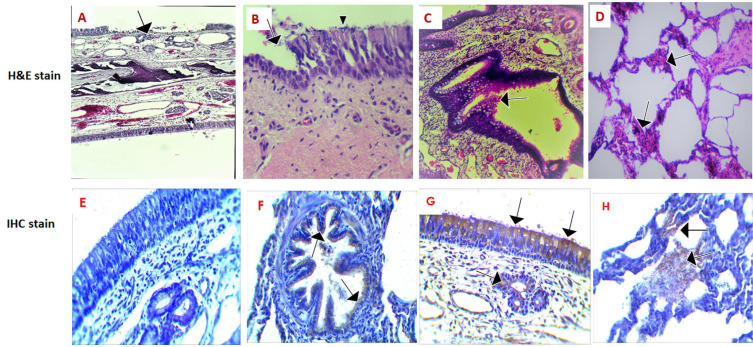
Histopathological and immunohistochemical changes in some MER-CoV naturally infected dromedary camels: (**A**): Nasal turbinate showed a denuded area of epithelial layer (arrow), H&E, ×100. (**B**): Bronchus showed a denuded area of epithelia (arrow) with many cells losing their cilia (arrowhead), H&E, ×400. (**C**): Nasal turbinate showed marked epithelial hyperplasia (arrow), H&E, ×100. (**D**): Lung showed interstitial pneumonia with alveolar wall thickening (arrow), H&E, ×100. (**E**): Nasal turbinate showed no IHC signal (negative control). (**F**): Bronchus showed detection of MER-CoV antigen in the epithelial layer and appears as a brown color (arrow). (**G**): Nasal turbinate showed IHC detection of MER-CoV antigen in the epithelial and glandular layer and appears as a brown color (arrows). (**H**): Lung showed detection of MER-CoV antigen in the thickened alveolar wall and appears as a brown color (arrow).

Histopathologic examination of the nasal turbinate, trachea, and bronchi sections stained with H&E stain revealed marked affections of the epithelial layer including the ciliated and mucous secreting cells. Frequent areas from these epithelia were denuded losing their apical surface (Figure 1A). Some of these cells that were normally ciliated appeared to lose their cilia (Figure 1B). Focal areas of hyperplasia were seen in the epithelial layers of both nasal turbinate and bronchi (Figure 1C). The H&E examination of the lungs of all the MERS-CoV infected animals showed acute interstitial pneumonia with thickening of the alveolar wall (Figure 1D). MERS-Co viral antigens were detected in these respiratory organs during immunohistochemical examination (Figure 1F–H) compared with the control negative section (Figure 1E).

### 2.3. Scanning Electron Microscopy (SEM) on Respiratory Organs Obtained from MERS-CoV Field Infected Animals 

Three major respiratory organs, the nasal turbinates, trachea, and lung, of some MERS-CoV infected animals under study were examined by the regular SEM technique. We examined several sections from these respiratory organs from the three infected animals and the two non-infected animals.

#### 2.3.1. Lesion Scoring and Pathological Alterations in the Nasal Turbinate of Some Field Infected Animals by the SEM 

**Figure 2:**
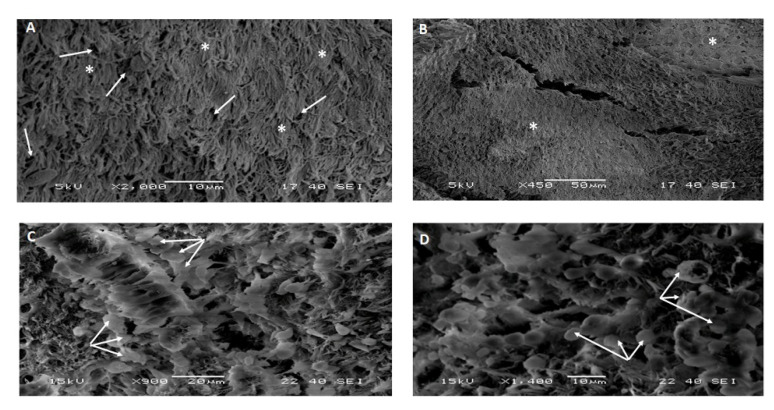
Scanning electron microscopy of the epithelium of the nasal turbinate of some MERS-CoV naturally infected dromedary camels compared to the non-infected animals: (**A**): Epithelium of nasal turbinate of a non-infected MERS-CoV dromedary camel showed the normal distribution of cilia giving the carpet shape appearance (asterisks) and normal goblet cells distribution across the section (arrows), ×2000. (**B**): Epithelium of Nasal turbinate of a positive MERS-CoV naturally infected dromedary camels showed an area of marked ciliary loss (asterisks) (×450). (**C**): Epithelium of nasal turbinate of a positive MERS-CoV naturally infected dromedary camel showed goblet cell proliferation (triple merged arrows), (× = 900). (**D**): Epithelium of nasal turbinate of a positive MERS-CoV naturally infected dromedary camel showed extensive goblet cell proliferation in higher magnification (triple merged arrows), ×1400.

In the ventral turbinate, the non-infected animals showing intact cilia representing what is called ‘the carpet-like appearance’ with a normal distribution of the goblet cells across the section (Figure 2 and Table 1). Animal number (1) showed a mild form of ciliary loss and severe goblet cell hyperplasia (GCH). Animal number (2) showed extensive ciliary loss with moderate goblet cell proliferation. Animal number (3) showed no loss of cilia and normal goblet cell distribution.

#### 2.3.2. Lesion Scoring and Pathological Alterations in the Trachea of Some Field Infected Animals by the SEM 

**Figure 3:**
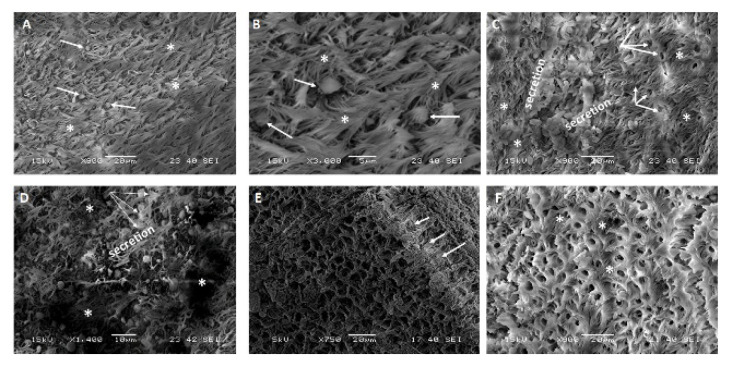
Scanning electron microscopy of the epithelium of the trachea of MERS-CoV naturally infected dromedary camels compared to the non-infected animals: (**A**): Epithelium of the trachea of a non-infected camel showed the normal distribution of cilia giving the carpet shape appearance (asterisks) and normal goblet cells distribution across the section (arrows), ×900. (**B**): Higher magnification of A (×3000). (**C**): The epithelium of the trachea of an infected camel showed areas of mild ciliary loss (asterisks), areas of ruptured proliferated goblet cell (triple merged arrows), and areas of mucous secretion (× = 900). (**D**): Epithelium of the trachea of an infected camel showed moderate ciliary loss areas (asterisks), moderate goblet cell proliferation areas (triple merged arrows) covered with mucous secretion (× = 1400). (**E**): Epithelium of the trachea of one infected camel showed complete damage of the apical portion of the luminal cell surface with complete ciliary loss and absence of the carpet shape appearance except for a small ciliated area on the upper right area of the scanning micrograph (arrows), ×750. (**F**): Epithelium of the trachea of one infected camel showed eroded epithelial proliferating cells interspersed with less ciliated areas (asterisks), ×900.

The trachea of the non-infected MERS-CoV animals showed the normal structure, including optimum distribution of cilia across all sections in addition to homogeneous goblet cell distribution (Figure 3 and Table 1). Animal number (1) showed neither ciliary loss nor goblet cell proliferation. Animal number (2) showed mild forms of ciliary loss and goblet cell proliferation. Animal number (3) showed moderate ciliary loss and goblet cell proliferation. Some sections showed eroded areas of the luminal surface epithelium.

#### 2.3.3. Alterations and some Pathological Findings in the Lung Tissues of Field Infected MERS-CoV Dromedary Camels by SEM 

**Figure 4:**
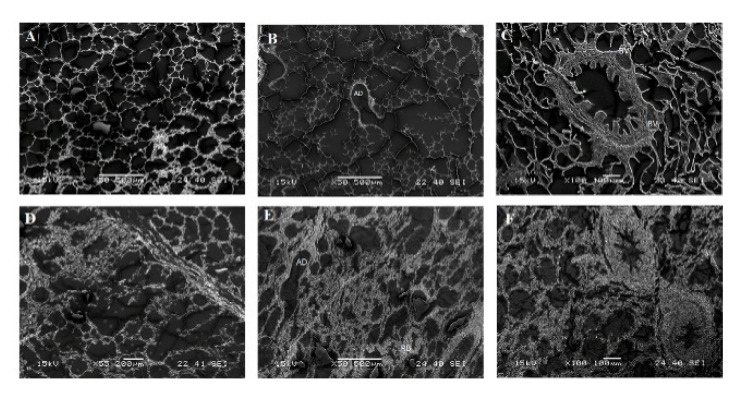
Scanning electron microscopy of the lungs of MERS-CoV naturally infected dromedary camels compared to the non-infected animals: (**A**): Lung tissues of a non-infected camel showed normal distribution of thin wall alveoli (×50). (**B**): Lung tissues of a non-infected camel showed normal distribution of thin wall alveoli and alveolar duct connected to the respiratory bronchioles (×50). (**C**): Lung tissues of a non-infected camel showed normal distribution of thin wall alveoli and normal bronchiole with two blood vessels (×100). (**D**): Lung tissues of an infected camel showed thickening of the walls of the alveoli as well as thickening of the inter-alveolar septa (×55). (**E**): Lung tissues of an infected camel showed thick walls of alveolar ducts (AD) connected to thick walls alveoli and respiratory bronchioles (RB), ×50. (**F**): Lung tissues of an infected camel showed thickening in the alveolar walls, inter-alveolar septa, and peribronchiolar tissue (×100).

The SEM examination of the left apical lobes of some lungs of the non-MERS-CoV infected animals showed the normal histological structure of the lung, including alveoli with thin walls and thin inter-alveolar septa (Figure 4). The presence of some alveolar ducts connected to the respiratory bronchioles was prominent in many sections. Terminal bronchioles showed a normal appearance. Simultaneously, the three MERS-CoV positive camels showed various degrees of lung pathology ranging from mild to severe interstitial pneumonia. The three infected animals showed thickening in the alveolar walls, inter-alveolar septa along thickening in the alveolar ducts and the respiratory bronchioles. The lumens of some terminal bronchioles contain a few cilia and mucous-secreting cells.

### 2.4. Identification of the MERS-CoV Lineage in Animals Admitted to This Abattoir

**Figure 5:**
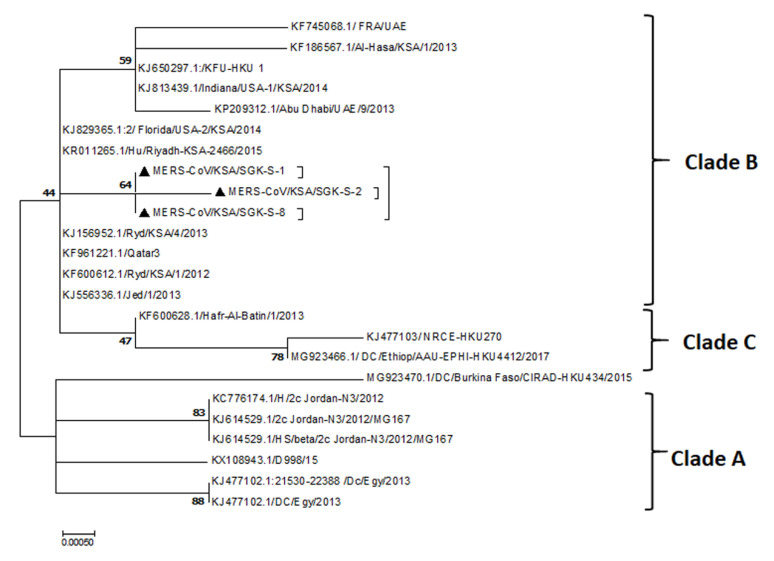
Identification of the circulating strains of MERS-CoV. The maximum likelihood analysis using the Mega-7 program of the partial MERS-CoV-S gene sequences from the nasal swabs of some dromedary camels received at this slaughterhouse. The obtained sequences (highlighted in black triangles) are clustered together with clade-B of MERS-CoV.

Our results showing the partial MERS-CoV-S gene sequences obtained from the nasal swabs of some of the dromedary camels from this abattoir are belong to the clade-B (Figure 5). 

## 3. Discussion

The dromedary camels remain the solely confirmed animal reservoir for the MERS-CoV. The comparison of the genomic sequences of many MERS-CoV isolates from the Arabian Peninsula and Africa revealed some insights about the evolution of the virus and the distribution of different clades per region [23,24,25]. Initially, clade A of MERS-CoV was circulating in dromedary camels and humans in these regions [26,27]. However, clade B is the main predominant clade across the Arabian Peninsula while clade C is mainly circulating in Africa [25]. The pathogenesis of the MERS-CoV in dromedary camels under field conditions remains largely unknown. Several studies conducted some experimental infections of very few numbers of animals [12,13,21]. Some other studies reported high detection rates of the MERS-CoV-RNAs in tissues from the lungs of dromedary camels in eastern Saudi Arabia [6]. Interestingly, most of the MERS-CoV experimental studies and surveillances conducted on members of the family Camelidae revealed mild clinical signs such as nasal discharge, rhinorrhea, and lacrimation [6,9,12,13,21,28]. However, the exact pathology induced by MERS-CoV in dromedary camels under the field infection using a scanning electron microscope is not well characterized yet. Some studies reported the normal structure of the respiratory tract in young and adult species of some animals, especially cattle, by the SEM; however, the histology of the respiratory tract of camels by SEM is still understudied. In a recent study, we showed some pathological alterations in the respiratory organs of three dromedary camels naturally infected with MERS-CoV by the H&E compared to two non-infected animals [22]. We first confirmed the detection of MERS-CoV-S and MERS-CoV-NC antigens in the tissues of these three animals by the IHC technique [22]. This was to ensure these tissues were infected with MERS-CoV. Further, we developed a lesion scoring based on various parameters of the H&E sections of these tissues. This scoring system revealed varying degrees of MERS-CoV affections in the respiratory organs of these animals [22]. In the present study, we used the same tissues to report any further pathological alterations that these three animals might have by the SEM. Our obtained results showing a marked consistency between the pathological changes under the light microscope using H&E stain with the SEM pictures of these organs.

This study revealed the formation of the ciliary network in the form of a ’‘carpet’ as an indicator of the normal respiratory structure, particularly in the trachea and lungs [29]. The ratio of the goblet cells to the number of ciliated cells may act as one of the important markers for healthy respiratory tissues [30]. One study showed a variable degree of loss of cilia in some nasal turbinate and trachea of some dromedary camels were shown under experimental infection by MERS-CoV following the administration of the recombinant MERS-CoV/Vaccinia virus- Ankara (MVA-S) [21]. 

In our study, three target animals that were MERS-CoV positive using two MERS-CoV targets by the real-time PCR as previously described [22] were slaughtered in parallel to the other two negative animals. Tissues from the respiratory organs, particularly the nasal turbinate, trachea, and lungs from these five animals, were processed and examined by the SEM. A lesion scoring system was developed based on two major parameters, including the ciliary loss and the goblet cell hyperplasia (Table 1, Figure 2 and Figure 3). We set up some parameters for our experiments to ensure the observed pathological changes are highly likely due to MERS-CoV natural infection. The positive naturally infected MERS-CoV dromedary camels were identified through two consecutive steps as suggested by the OIE. First, we applied the quick Ag detection test to do a quick screening of a large number of animals to identify some positive animals. Second, the positive animals were subjected to real-time PCR assay using two targets (ORF1a and UpE) genes as suggested by the WHO. Third, we selected animals having the lowest Ct values less than or equal to16 to ensure high viral load in the respiratory passages of these animals. Fourth, we used some PCR negative dromedary camels as a negative control. The lesions were described only if animals were both PCR and IHC-positive using specific MERS-CoV antibodies. Besides, these animals neither showed any H&E pathological and histopathological finding related to bacterial infections as suppurative bronchopneumonia, polymorph leukocytic aggregations. Also, animals that showed any signs of hemorrhagic pneumonia due to the slaughtering technique practiced in KSA were excluded. The animal showed any suppurative or hemorrhagic pneumonia were not considered in this study. Meanwhile, we included two negative animals (Ag detection kits, PCR using two targets, and IHC using different MERS-CoV antibodies). Based on our previous publication, presence of the interstitial pneumonia in all positive MERS-CoV infected animals as confirmed by PCR and IHC motivated us to explore the lesions by the SEM.

In this scoring system, we examined several sections per organ per animal. In each section, we examined at least 15 fields under the SEM. We reported the average score per each organ per each animal (Table 1, Figure 2 and Figure 3). The three positive animals showed a variable degree of ciliary loss in both the nasal turbinate and trachea. The variations in the degree of ciliary loss could be attributed to some factors such as the magnitude of viral infection/viral load per each animal and the stage of the viral infection per each animal. The virus load was correlated to the severity of the SARS-CoV-2 infections in humans as well as to the outcomes of the infected individuals [31]. Animals (1, 2, and 3) showed a mild, severe, and absence of ciliary loss in the nasal turbinate, respectively. However, animals (1, 2, and 3) showed absence, mild, and moderate ciliary loss in the trachea of these animals, respectively. Our results are very much consistent with the earlier study that used the chimeric MERS-CoV-S1 recombinant antigen to challenge some vaccinated camels [21]. These data are also confirming the tropism and localization of the MERS-CoV in some respiratory organs, particularly the nasal turbinate and lung tissues [32]. That is the most suitable sample for the diagnosis of MERS-CoV is the nasopharyngeal swabs, particularly those collected from the back of the nose close to the nasal turbinate [3]. 

Some viral infections have negative impacts on the function of cilia in respiratory organs [33]. Thus, we believe the ciliary impermanent and loss is one of the characteristic pathological findings during MERS-CoV infections in dromedary camels. The goblet cell hyperplasia (GCH) was reported on many occasions in response to viral infections, inflammations, and in some types of cancers [34]. The respiratory syncytial virus infects the respiratory tissues, particularly the ciliated cells, and induces GCH in humans [35]. Both the SEM and the transmission electron microspores (TEM) are used for the diagnosis of SARS-CoV infections in various organs of the infected patients. Thickening in the inter-alveolar septa and infiltration of some leukocytes was reported in our study, which is very much consistent with some studies on SARS-CoV-2 infection in humans [18]. The most common feature of the pathological alterations in the lungs noted is the signs of interstitial pneumonia of various degrees in the three positive animals. The main notable findings are the thickening in the inter-alveolar septa due to marked leukocyte infiltrations, particularly lymphocytes and macrophages [35]. Based on the lesion scoring using the H&E, the antigen detection, and the SEM observations in these three animals, we conclude that the observed pathological alterations in the respiratory organs of these animals are most likely due to the MERS-CoV infection. This pattern is very much consistent with viral pneumonia in camels reported earlier [36]. However, there are no characteristic pathognomonic lesions reported for MERS-CoV and SARS-CoV-2 infections in the lungs. Further large-scale research studies are required for revealing the most common pathological changes triggered by the field MERS-CoV infection in camels.

## 4. Materials and Methods 

### 4.1. Animal Selection 

A molecular surveillance study among dromedary camels in one of the central regional slaughterhouses in the central region was conducted [17]. Based on this study, we selected three MERS-CoV infected dromedary camels as well as two negative animals as negative controls. Briefly, we examined the animals for MERS-CoV antigen using the BIONOTE® Rapid MERS-CoV Ag Test Kit as per the kit’s instructions. Positive animals were quarantined in a separate place, and their collected nasal swabs were tested by real-time PCR as previously described [3,22]. 

The three positive animals had the lowest Ct values of the tested animals (average Ct values =14). Details about animals used in this study can be found in Table 1. These five animals were slaughtered by the standard protocols, as previously described [17]. We collected some respiratory organs, as shown below, for further SEM examination.

### 4.2. Histopathological Examination

The selected animals were subjected to necropsy examination after being positive by real-time PCR. Tissues from the upper and the lower respiratory tract were fixed in 10% neutral-buffered formalin immediately after slaughtering for five days. The tissues were processed with a thickness of 4 um, and paraffin sections were stained with Hematoxylin and Eosin (H&E) for histopathological examination as previously described by Bancroft and Cook (1994).

### 4.3. Immunohistochemistry

Tissue sections from the trachea, nasal turbinates, and lungs were screened for the presence of the MERS-CoV-S1 and MERS-CoV-N by immunohistochemistry. We used the polyclonal rabbit anti-MERS-CoV-S protein, S1 (Sinobiological, Wayne, PA 19087, USA), and monoclonal mouse anti-MERS-CoV Nucleocapsid (NC), (Sinobiological, catalog#40068-V08B, AFS88943.1, Met1-Asp413) to detect the MERS-CoV-S and MERS-CoV-N proteins, respectively. The technique was carried out as per the manufacturer’s instructions (Technicon’s LTD, Tokyo, Japan).

### 4.4. RNA Extraction, Real-Time PCR, and Sequencing of MERS-CoV-S Gene

The viral RNAs were extracted from the nasal swabs of dromedary camels admitted to this slaughterhouse in the central region of Saudi Arabia. The procedures of the RNA extraction were conducted as previously described [37]. Testing of the nasal swabs for the MERS-CoV using two targets (ORF1a and UpE) was carried out as previously described [37,38,39,40]. Sequencing of the MERS-CoV-S gene was carried out using the Ion Torrent-NGS sequencing technology. Assembly and annotation of the obtained sequences was carried out as previously described [41]. The phylogenetic analysis and clade identification was carried out by the MEGA-X software as previously described [42].

### 4.5. Scanning Electron Microscope

We prepared some 10% buffered formaldehyde-fixed tissues from both the natural infected MERS-CoV camels and non-infected negative control animals (the nasal turbinate, the trachea, and the lungs) to be examined under the scanning electron microscope (SEM). This technique was performed as described elsewhere [24]. Briefly, all sections were cut from the paraffin wax blocks into thin sections (10 um thickness) using the microtome. The lung tissues were kept in a solution of 3% Osmic acid for two hours at 4°C. Washing of these tissues three times was performed by the phosphate buffer saline. The dehydration of the tissues was conducted in increasing gradient ethanol solutions starting with 30%, 50%, 70%, 10%, and finally 100%. Each dehydration step was incubated for 15 min. We removed the ethanol, then mount the tissues with the hexamethyldisilazane (HMDS) for 5 min, then removed. We added a fresh HMDS to the tissues then incubated them at room temperature at the fume hood. We mounted the samples into the SEM stubs, sputter coat (gold). Tissue sections were examined under the scanning electron microscope (JOEL, JSM-5510LV, Japan) using the SEM Control User interface Version 5.04, JOEL TECHNOLOGIES LIMITED, Japan). To establish the lesion scoring system for various respiratory organs, we examined several sections per each organ. We examined an average of 15 fields per organ to take the average scoring. We examined each section at three different magnification power (low, medium, and high). 

## Figures and Tables

**Table 1:** Lesion scoring of the nasal turbinate and trachea of some dromedary camels naturally infected with MERS-CoV based on SEM findings.

Organ	Nasal Turbinate			Trachea		
Number of animal	1	2	3	1	2	3
Ciliary loss	+	+++	-	-	+	++
Goblet cell hyperplasia	+++	++	-	-	+	++

(-) no ciliary loss or goblet cell proliferation; (+) mild ciliary loss or goblet cell proliferation, (++) moderate ciliary loss or goblet cell proliferation; (+++) severe ciliary loss or goblet cell proliferation.

## Data Availability

All data are presented in the manuscript.
